# *Bradyrhizobium japonicum* IRAT FA3 promotes salt tolerance through jasmonic acid priming in *Arabidopsis thaliana*

**DOI:** 10.1186/s12870-022-03977-z

**Published:** 2023-01-30

**Authors:** Melissa Y. Gomez, Mercedes M. Schroeder, Maha. Chieb, Nathan K. McLain, Emma W. Gachomo

**Affiliations:** Department of Microbiology and Plant Pathology, University of California Riverside, Riverside, CA 92507 USA

**Keywords:** Plant growth promoting rhizobacteria, Salt stress, *JAR1*, *MYC2*, Reactive oxygen species

## Abstract

**Background:**

Plant growth promoting rhizobacteria (PGPR)**,** such as Bradyrhizobium *japonicum* IRAT FA3, are able to improve seed germination and plant growth under various biotic and abiotic stress conditions, including high salinity stress. PGPR can affect plants’ responses to stress via multiple pathways which are often interconnected but were previously thought to be distinct. Although the overall impacts of PGPR on plant growth and stress tolerance have been well documented, the underlying mechanisms are not fully elucidated. This work contributes to understanding how PGPR promote abiotic stress by revealing major plant pathways triggered by *B. japonicum* under salt stress.

**Results:**

The plant growth-promoting rhizobacterial (PGPR) strain *Bradyrhizobium japonicum* IRAT FA3 reduced the levels of sodium in *Arabidopsis thaliana by 37.7%*. *B. japonicum* primed plants as it stimulated an increase in jasmonates (JA) and modulated hydrogen peroxide production shortly after inoculation. *B. japonicum*-primed plants displayed enhanced shoot biomass, reduced lipid peroxidation and limited sodium accumulation under salt stress conditions. Q(RT)-PCR analysis of JA and abiotic stress-related gene expression in Arabidopsis plants pretreated with *B. japonicum* and followed by six hours of salt stress revealed differential gene expression compared to non-inoculated plants. *Response to Desiccation* (*RD*) gene *RD20* and reactive oxygen species scavenging genes *CAT3* and *MDAR2* were up-regulated in shoots while *CAT3* and *RD22* were increased in roots by *B. japonicum*, suggesting roles for these genes in *B. japonicum*-mediated salt tolerance. *B. japonicum* also influenced reductions of *RD22*, *MSD1*, *DHAR* and *MYC2* in shoots and *DHAR*, *ADC2*, *RD20*, *RD29B*, *GTR1*, *ANAC055*, *VSP1* and *VSP2* gene expression in roots under salt stress.

**Conclusion:**

Our data showed that *MYC2* and *JAR1* are required for *B. japonicum*-induced shoot growth in both salt stressed and non-stressed plants. The observed microbially influenced reactions to salinity stress in inoculated plants underscore the complexity of the *B. japonicum* jasmonic acid-mediated plant response salt tolerance.

**Supplementary Information:**

The online version contains supplementary material available at 10.1186/s12870-022-03977-z.

## Background

The impacts of abiotic stress factors, e.g. salt, are far-reaching with respect to crop production [1]. Excessive salt causes oxidative and osmotic stress, induces production of reactive oxygen species (ROS), reduces the photosynthetic rate, affects normal plant development, interferes with ionic balances and nutrient availability and alters soil pH, water availability and microbial diversity [2]. Methods employed to reduce soil salinity include soil flushing, liming, plant breeding and genetic engineering of salt tolerant crops [3]. Such methods have had limited success, are time consuming, expensive and can cause genetic erosion of indigenous crops species [3].

In order to cope with abiotic stress, plants employ several physical structures, e.g., a cuticle layer to reduce moisture loss and shield against incoming stressors [1] and regulatory molecular mechanisms [2, 4]. Abiotic stress alters cell homeostasis and increases production of ROS [5, 6]. Plants counter the increase in ROS through their defense mechanisms, which involve antioxidants and ROS-scavenging enzymes [4–6]. These enzymes include superoxide dismutase (SOD), ascorbate peroxidase (APX), catalase (CAT), glutathione peroxidase (GPX) and peroxiredoxin (PrxR) [7, 8]. The Salt Overly Sensitive (SOS) pathway has also been shown to play a role in mediating ionic homeostasis at the cellular level in Arabidopsis [7].

Plant growth-promoting rhizobacteria (PGPR) are known to alleviate various abiotic and biotic stresses that plants may encounter [9, 10]. Some PGPR have been reported to increase the expression of antioxidant enzymes in plants under salt stress, e.g. *Dietzia natronolimnaea* STR1 in wheat [9]. *Bradyrhizobium japonicum* strains and other PGPR have been reported to enhance plant growth and productivity [8, 11], to induce production of phytohormones such as indole-3-acetic acid, zeatin, gibberellic acid, ethylene and abscisic acid [12], and to promote tolerance to drought [8] and salinity [9].

PGPR microbe-associated molecular patterns (MAMPs), and their specific cognate plant receptors, have evolved to elicit a more transient and less robust plant response than that provoked by pathogenic microbial MAMPs [13]. The plant response to PGPR MAMPs has been shown to include extracellular alkalinization, a cellular Ca^2+^ increase, oxidative burst and up-regulation of pathogenesis-related genes [13, 14]. Oxidative burst is the rapid production of ROS, i.e., molecules such as superoxide (O_2_^−^) or hydrogen peroxide (H_2_O_2_), that can react with a wide range of biomolecules resulting in signal transduction or cellular damage [15]. ROS-mediated signaling is associated with the regulation of various biological processes, including biotic and abiotic stress responses [4, 6, 13]. In *Vinus vinifera* cells treated with a PGPR MAMP, the oxidative burst and typical defense marker gene induction were observed to be weaker and more transient than those elicited by pathogen MAMPs [13]. These plant responses to a PGPR MAMP were indicative of the onset of priming, as further evidenced by a significant reduction in disease when leaves were subsequently challenged with a necrotrophic fungus [13, 16].

Inoculation of plants by PGPR induces a physiological state in which plants are potentiated for defense responses and have an enhanced resistance to abiotic stress [6]. Several PGPR have been assessed with regard to salt stress amelioration and have been found to help plants overcome mild salt stress [9, 17]. Inoculating wheat crops with *Arthrobacter protophormiae* and *D. natronolimnaea* resulted in increased shoot and root weight and length under salt stress [9]. Treatment of plants with PGPR has been shown to increase salt tolerance through the SOS pathway [9, 17]. Several factors have been shown to prime plants e.g. salicylic acid (SA), SA synthetic analogs, abscisic acid (ABA), pathogen-associated molecular patterns (PAMPs), PGPR, plant growth promoting fungi (PGPF), and certain chemicals [18, 19]. Priming by PGPR has been extensively studied in association with induced systemic resistance (ISR), where pretreatment of plants with PGPR reduces disease in the plant [16]. ISR induction in Arabidopsis by the PGPR *Pseudomonas fluorescens* WCS417r has been shown to act through the jasmonic acid (JA) and ethylene (ET) signaling pathways [20]. However, this induced systemic resistance was not accompanied by induction of JA or ET production in the primed plants [21]. ISR induced by the PGPR *Bacillus subtilis* GB03 is independent of SA, JA pathways, but requires ET signaling [22]. Nevertheless, *B. subtilis* GB03 significantly up-regulated defense mechanisms mediated by SA and JA, indicating that the mechanism of ISR can vary among different PGPR.

Limited information is available on the genetic mechanisms through which PGPR stimulate salt tolerance. In our previous studies we observed that *B. japonicum* altered root architecture by regulating auxin transporters [23] and improved drought stress tolerance [8] and therefore we decided to investigate its role in salt stress tolerance. The objective of this study was to determine how *B. japonicum* affects the salt stress response of Arabidopsis and to uncover the molecular mechanism involved. Our results indicate that *B. japonicum* IRAT FA3 improves tolerance to salt stress by impacting biomass, root architecture, oxidative stress, and transcriptional regulation of stress response pathways. We found that *B. japonicum* induced tolerance against salt stress through modulation of jasmonic acid production and JA responsive genes, ROS, and upregulation of salt stress-related *Responsive to Desiccation (RD)* genes.

## Results

### *Bradyrhizobium japonicum* IRAT FA3 affects Arabidopsis shoot and root growth under salt stress

Experimental parameters were determined using wild type, Col-0, Arabidopsis seedlings grown on agar plates with and without additional sodium chloride (NaCl, salt) (Supplementary Fig. S1). Shoot weight significantly decreased in plants when 100 mM NaCl was added to the growth medium, while the 200 mM concentration was plant-lethal (Supplementary Fig. S1A). When we assayed for the number of *B. japonicum* colony forming units (CFU) in liquid culture under salt stress, we observed that addition of NaCl to the liquid culture at a final concentration of 100 mM had no significant effect on the number of CFUs when compared to the rate in commercially available LB (Supplementary Fig. S2A). However, 200 mM and 300 mM significantly impacted bacterial growth. No significant differences in the root colonization of Arabidopsis by *B. japonicum* were observed between control and plants treated with 100 mM NaCl (Supplementary Fig. S2B). Therefore, 100 mM NaCl was determined to be an appropriate concentration for salt stress treatment in this study.

**Fig. 1:**
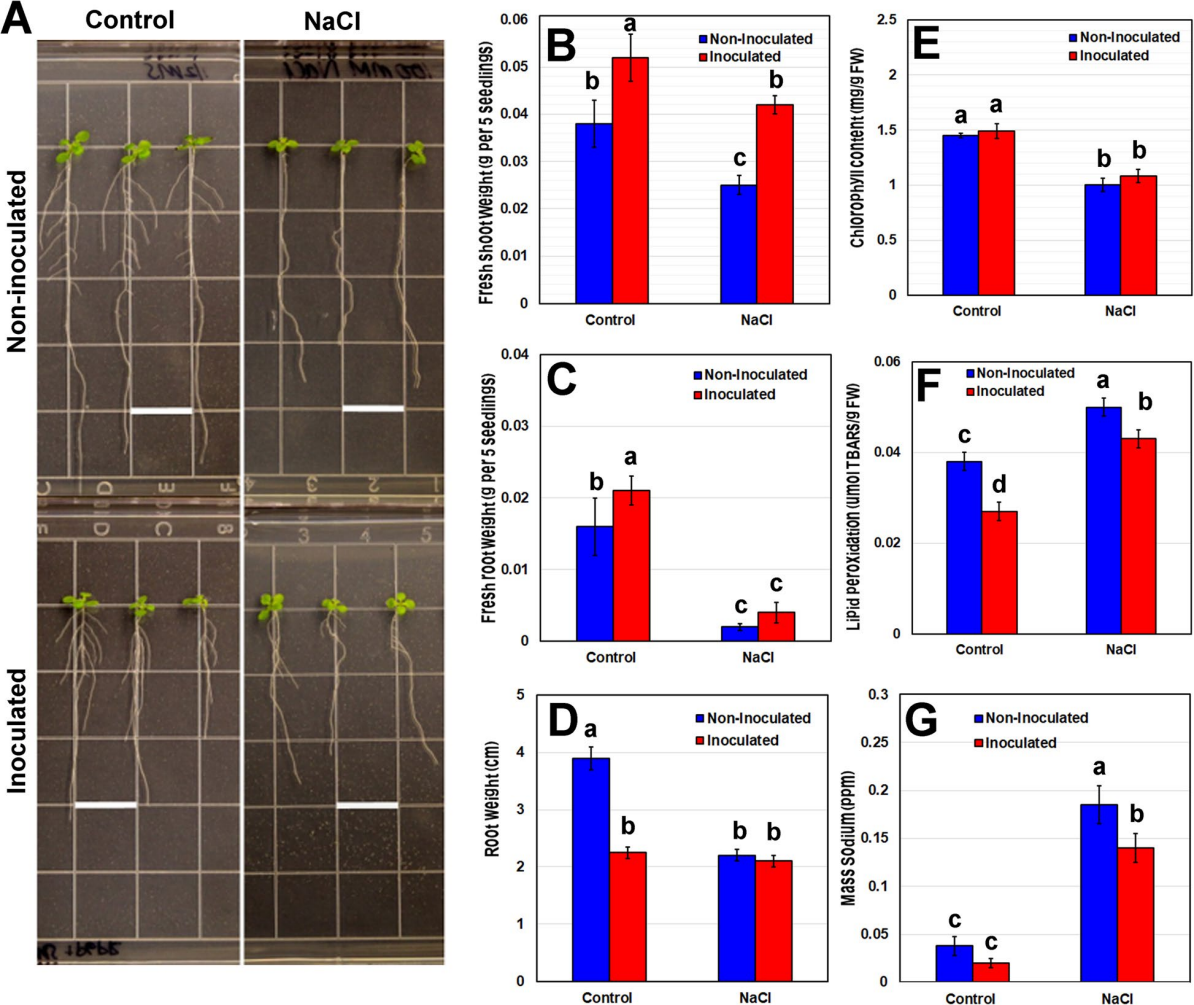
Effects of *B. japonicum* on plant growth under salt stress. Arabidopsis developmental phenotypes were analyzed in response to salt stress, in the presence (inoculated) or absence (non-inoculated) of *B. japonicum*. **A** Representative images of whole seedlings display root growth differences, while graphs show (**B**) shoot biomass, (**C**) root biomass, and (**D**) new primary root growth comparisons between treatments. Measurements of (**E**) total chlorophyll and (**F**) malondialdehyde (MDA) (**G**) sodium (ppm) content in the shoot tissue of Arabidopsis after fourteen days of co-cultivation with *B. japonicum* and seven days of salt (100 mM NaCl) stress or control treatment. Data are the mean ± standard error with different letters indicating significant differences. Scale bars indicate 1.3 cm; ANOVA-Tukey, *p* < 0.05

Plant growth and development was reduced by high salinity, but co-cultivation with *B. japonicum* minimized the effects of salt stress (Fig. 1A, B). *B. japonicum* mitigation of salt stress effects varied with the growth parameter measured. In *B. japonicum*-inoculated plants, high salinity significantly reduced shoot weight and root weight but not root length (Fig. 1B-D). The *B. japonicum*-stimulated increase in shoot weight was observed in non-stressed and salt-stressed plants (Fig. 1B). However, *B. japonicum* did not increase root weight compared to uninoculated control plants under salt (Fig. 1C). Salt stress played a significant role in contributing to root biomass reduction. Salinity stress also shortened primary root growth by 22% in non-inoculated plants, to lengths matching those of inoculated no-stress plants (Fig. 1D). However, the new root growth in *B. japonicum* treated roots was not significantly different in non-stressed and salt stressed plants (Fig. 1D). Relative to uninoculated plants, *B. japonicum* co-cultivation significantly decreased the primary root growth by 37% under non-stress conditions (Fig. 1D).

### B. japonicum decreased lipid peroxidation under salinity stress while chlorophyll content remained unaffected.

Surprisingly, inoculating plants with *B. japonicum* did not alter the chlorophyll content in non-stressed and salt stressed plants. High salinity significantly decreased chlorophyll content in both *B. japonicum*-inoculated and non-inoculated plants by nearly 30% (Fig. 1E) indicating that chlorophyll production and possibly photosynthesis were negatively impacted by salt stress in both inoculated and non-inoculated plants. MDA is a byproduct of lipid peroxidation and the degree of lipid peroxidation in tissues can be estimated by the amount of MDA present [24]. MDA is the dominant compound measured with the thiobarbituric acid reactive substances test (TBAR), in which the lipid peroxidation products react with thiobarbituric acid (TBA) to yield a pink chromagen called TBARS [25]. In our study, MDA increased by 46.8% under high salinity in non-inoculated plants (Fig. 1F). *B. japonicum* inoculation significantly lowered the lipid peroxidation indicator under both non-stressed and salt stress conditions relative to uninoculated plants (Fig. 1F), indicating that *B. japonicum* plays a role in alleviating oxidative stress caused by high salinity.

### B. japonicum decreases sodium accumulation in plants.

Measurement of the total sodium content revealed no significant differences between inoculated and uninoculated plants under normal conditions. However, *B. japonicum* inoculation significantly reduced sodium accumulation under salt stress. In co-inoculated plants grown on 100 mM NaCl for fourteen days, *B. japonicum* reduced total sodium levels in whole plant tissue by 37.7% relative to the uninoculated salt-stressed plants (Fig. 1G).

### B. japonicum influences H_2_O_2_ levels and RBOH gene expression in Arabidopsis

**Fig. 2:**
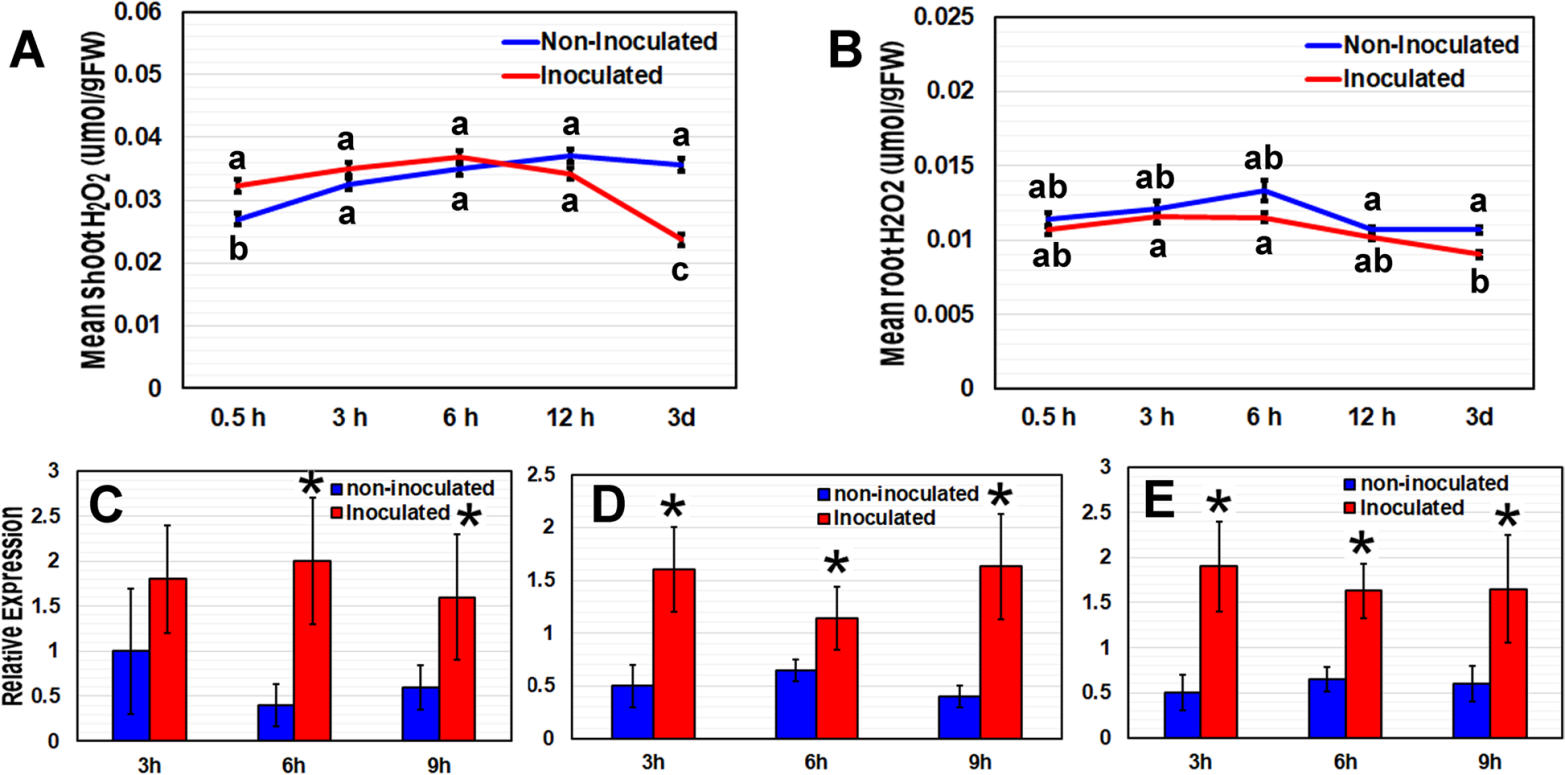
Arabidopsis H_2_O_2_ levels and *RBOH* gene expression in response to *B. japonicum*. H_2_O_2_ levels in (**A**) shoot and (**B**) root tissues of *B. japonicum*-inoculated and non-inoculated plants at time points ranging from 0.5 h (hr) to 3 days (d). Data are the mean ± standard error with different letters representing significant differences; ANOVA, Tukey, p < 0.05. Arabidopsis(**C**) *RBOHD,* (**D**) *RBOHE* and (**E**) *RBOHF* gene expression in whole plant tissue at 3, 6 and 9 h post inoculation (hpi) after co-cultivation with *B. japonicum*. qRT-PCR measurements indicate mean expression levels relative to the *IPP2* reference gene ± standard error with asterisks representing significant differences from the corresponding control; Student’s *t*-Test, *p* < 0.05

*B. japonicum*-inoculated plants exhibited a 22% increase in H_2_O_2_ content in shoot tissue at 0.5 h post inoculation (hpi) compared to non-inoculated plants (Fig. 2A). Levels of H_2_O_2_ in *B. japonicum*- inoculated plants were not significantly different from levels in the control plants between 3–12 hpi. At 3 days post inoculation (dpi) the H_2_O_2_ levels in the shoots of *B. japonicum*-inoculated plants decreased by 27%. In the roots, no significant differences in H_2_O_2_ levels were seen between inoculated and non-inoculated plants, except at 3 dpi when the levels were significantly lower in *B. japonicum*-co-cultivated plants (Fig. 2B). The fluctuations of H2O2 observed in the non-inoculated plants can be attributed to the natural fluctuations patterns that are dependent on an intrinsic circadian rhythm.

Several studies have shown that early plant responses to PGPR can include *Respiratory Burst Oxidase Homolog* (*RBOH*) gene expression that leads to ROS production [26, 27]. *RBOHD*, *RBOHE* and *RBOHF* are known to be expressed in response to biotic and abiotic stress [27, 28]. Gene expression analysis after *B. japonicum* inoculation revealed significant increases in *RBOHE* and *RBOHF* expression at 3, 6 and 9 hpi, and in *RBOHD* at 6 and 9 hpi, relative to non-inoculated plants (Fig. 2CDE). Elevated *RBOH* expression indicated that *B. japonicum* stimulated ROS production in the plants soon after inoculation. Increase in H_2_O_2_ production results in priming of plants against biotic and biotic stress [13, 16].

### B. japonicum differentially affects ROS scavenging enzymes under salinity stress.

**Fig. 3:**
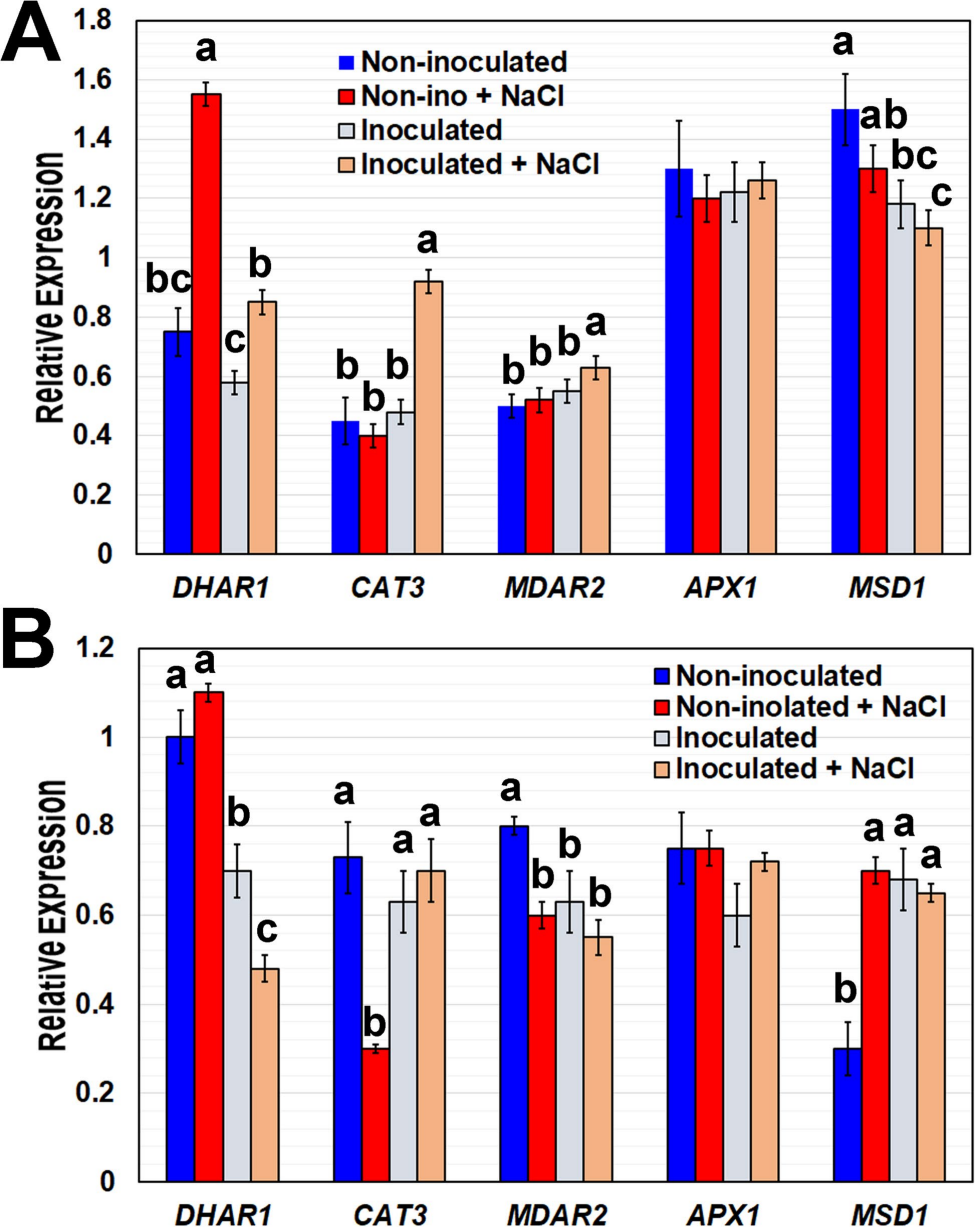
Transcriptional levels of the hydrogen peroxide detoxifying enzymes after *B. japonicum* inoculation and salt treatment. Plants were inoculated with *B. japonicum* and after 7 days they were treated with 100 mM NaCl for 6 h before sample collection. qRT-PCR analysis of ROS scavenging enzymes from non-inoculated and *B. japonicum*-inoculated root tissue, with and without 100 mM NaCl treatment in (**A**) shoot and (**B**) root tissues. Graphs show expressions of *Dehydroascorbate Reductase 1* (*DHAR1*), *Catalase 3* (*CAT3*), *Monodehydroascorbate Reductase 2* (*MDAR2*), *Ascorbate Peroxidase 1* (*APX1)* and *Manganese Superoxide Dismutase* (*MSD1*) relative to the *IPP2* reference gene ± standard error, with different letters representing significant differences separately for each gene, ANOVA, Tukey, *p* < 0.05

We determined the expression of genes encoding ROS scavenging enzymes six hours after high salinity (100 mM NaCl) treatment, which was preceded by one week of co-cultivation with *B. japonicum*. The antioxidant enzymes, *Manganese Superoxide Dismutase 1* (*MSD1*, At3g10920), *Catalase 3* (*CAT3*, At1g20620), *Ascorbate Peroxidase 1* (*APX1*, At1g07890), *Dehydroascorbate Reductase* (DHAR, At1g19570), and *Monodehydroascorbate Reductase 2* (*MDAR2*, At5g03630), are involved in scavenging excess hydrogen peroxide (H_2_O_2_) in the cell [5, 6, 29]. Salt stress increased H2O2 production in plants (data not shown). In non-inoculated plants, salt stress reduced *CAT3* and *MDAR2* and increased *MSD1* expression in root tissue and *DHAR* expression in shoot tissue. Salt stress had no effect on *CAT3*, *MDAR2*, or *MSD1* expression in shoot tissue or on *DHAR* in root tissue (Fig. 3). These results corroborated the data by Kilian et al. (2007) [30]. Under salt stress, *B. japonicum increased* the expression of *CAT3* in roots and shoots, relative to non-inoculated plants (Fig. 3). *B. japonicum* co-cultivation also influenced *MDAR2* and *MSD1* expression in shoots under salt stress, increasing and decreasing each, respectively (Fig. 3). *APX1* expression was not significantly altered by salt stress or bacterial co-cultivation (Fig. 3). Antioxidant expression levels were largely similar between all *B. japonicum* inoculated (non-stressed and salt-stressed) plants, with the exception of *CAT3* and *MDAR2* in shoots (Fig. 3). Under non-stressed conditions, *B. japonicum* downregulated the expression of *MSD1* in shoot tissue and *DHAR* in root tissue. Conversely, *B. japonicum* upregulated *MSD1* gene expression in root tissue. These data indicate that ROS regulation in response to salt stress and *B. japonicum* inoculation is complex. *B. japonicum*’s influence under non-stressed condtitions varied depending on the gene and plant tissue. Some *B. japonicum-*driven changes in gene expression were only present under salinity stress as in the case of *CAT3, MDAR2*, and *DHAR* in shoot tissue and *CAT3* root tissue. Interestingly, the expression of *MSD1* and *MDAR2* in shoots and roots after *B. japonicum* treatment followed the same trend as after salt stress.

### *B. japonicum* induces jasmonate production in Arabidopsis

**Fig. 4:**
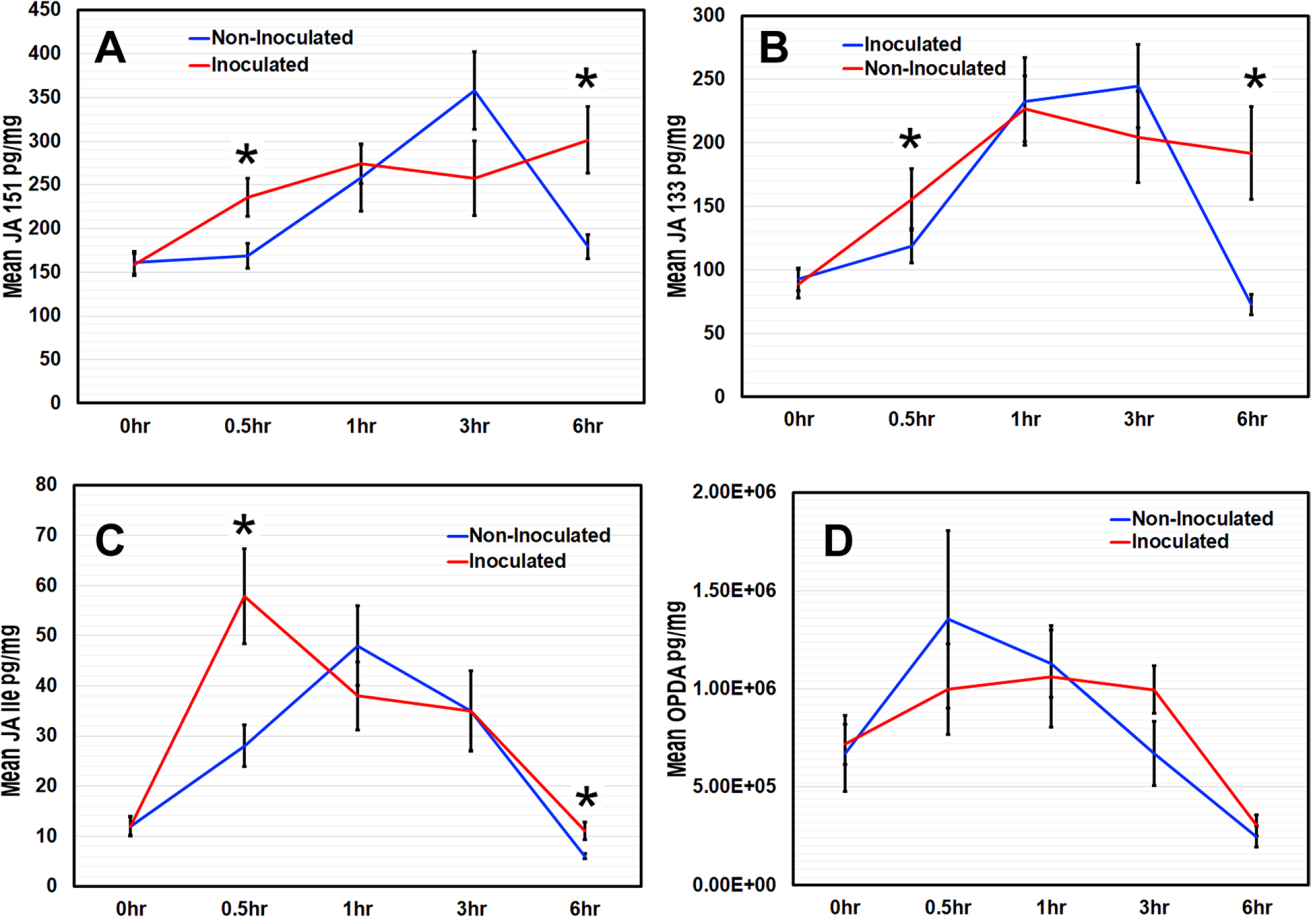
*B. japonicum* influences jasmonate production in Arabidopsis. Arabidopsis seedlings were treated with *B. japonicum* (inoculated) or 10 mM MgSO_4_ (non-inoculated) for up to 6 h. (**A**) JA 151, (**B**) JA 133, (**C**) jasmonoyl-isoleucine (JA-Ile) and (**D**) JA precursor 12-oxophytodienoic acid (OPDA) levels were measured by liquid chromatography mass spectrometry (LCMS). Graphs show mean data ± standard error with asterisks representing significant differences from the corresponding non-inoculated control; Student’s *t*-test, *p* < 0.05

We measured jasmonate (JA) (Fig. 4) and abscisic acid (ABA) (Supplementary Fig. S3) levels in plants with and without *B. japonicum-*cocultivation, for up to 6 h, using liquid chromatography mass spectrometry (LCMS).

Treatment with *B. japonicum* led to increased production of jasmonates: JA-isoleucine (JA-Ile), 12-oxophytodienoic acid (OPDA), and JA 151 and JA 133 (prominent mass spectra peaks that are characteristic of methyl jasmonate in mass spectrometry) [30, 31] in plants at 0.5 and 6 hpi (Fig. 4). *B. japonicum* induced an increase in JA soon after inoculation and then lowered it to the levels found in uninoculated plants. All jasmonates were significantly higher in *B. japonicum*-inoculated plants at 6 hpi except for OPDA levels, which dropped to below those in non-inoculated plants after 1 hpi. ABA production was not altered by *B. japonicum* co-cultivation (Supplementary Fig. S3).

**Fig. 5:**
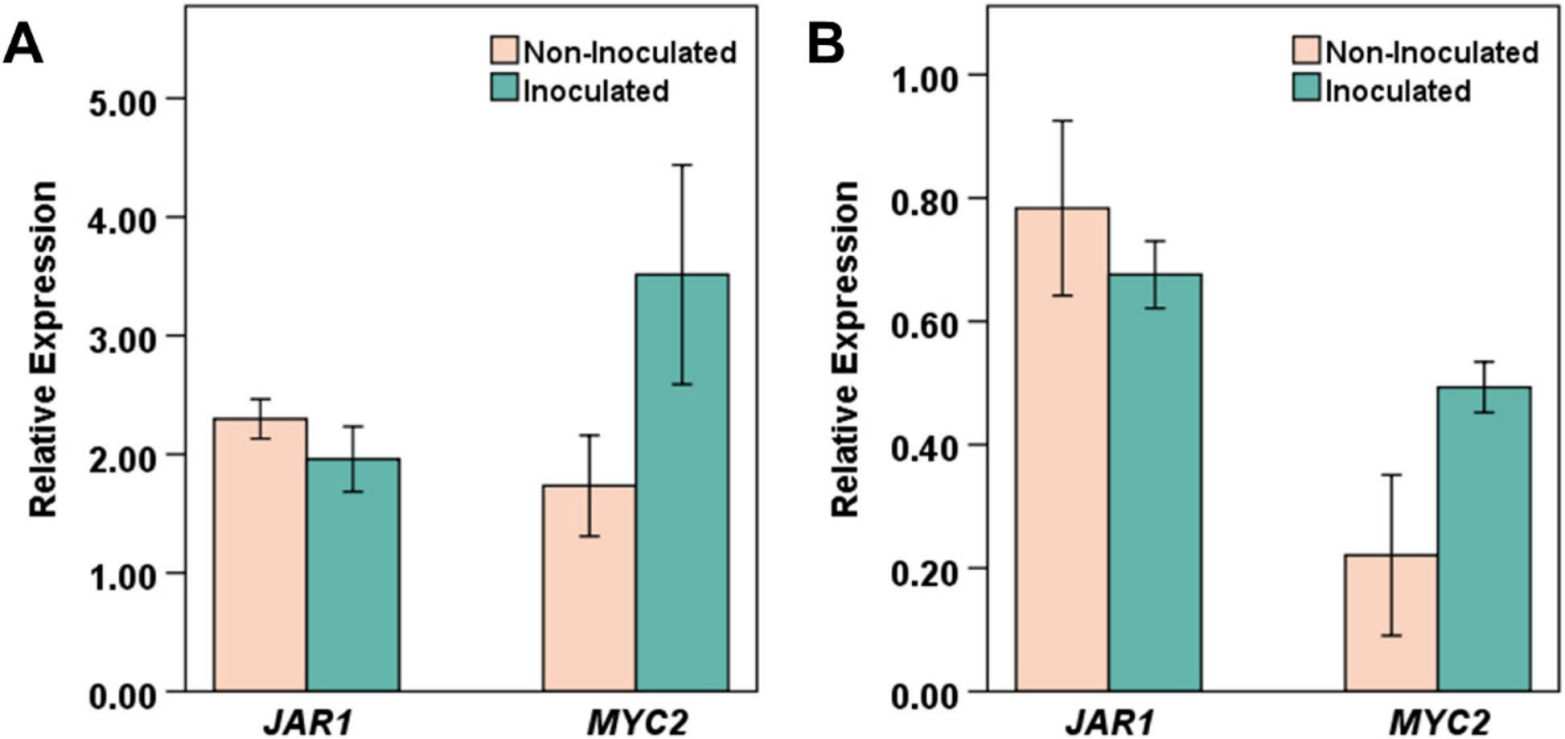
Early JA-related gene expression in response to *B. japonicum* co-cultivation. Arabidopsis seedlings treated with *B. japonicum* (inoculated) or 10 mM MgSO_4_ (non-inoculated) for 0.5 h. Graphs show expression of *JAR1* and *MYC2* in (**A**) shoot (*JAR1; p* = 0.332; *MYC2; p* = 0.112) and (**B**) root (*JAR1; p* = 0.504; *MYC2; p* = 0.070) tissues. Mean expression relative to the *IPP2* reference gene ± standard error. Student's *t*-Test, *p* < 0.05

After observing the *B. japonicum*-induced increase in jasmonate production, we decided to determine the expression of key JA pathway genes, *JAR1* and *MYC2*. *JAR1* (At2g46370) encodes a jasmonoyl-isoleucine synthetase that catalyzes the formation of JA-Ile [32] while *MYC2* (At1g32640) is a major hub in jasmonate signaling pathways, a regulator of ISR, and a regulator of crosstalk between ABA, JA, salicylic acid, and auxin pathways during plant development [33–35]. Although no statistically significant differences were observed in *JAR1* and *MYC2* expression levels between inoculated and non-inoculated plants at 0.5 hpi, a slight up-regulation of *MYC2* was apparent (Fig. 5).

**Fig. 6:**
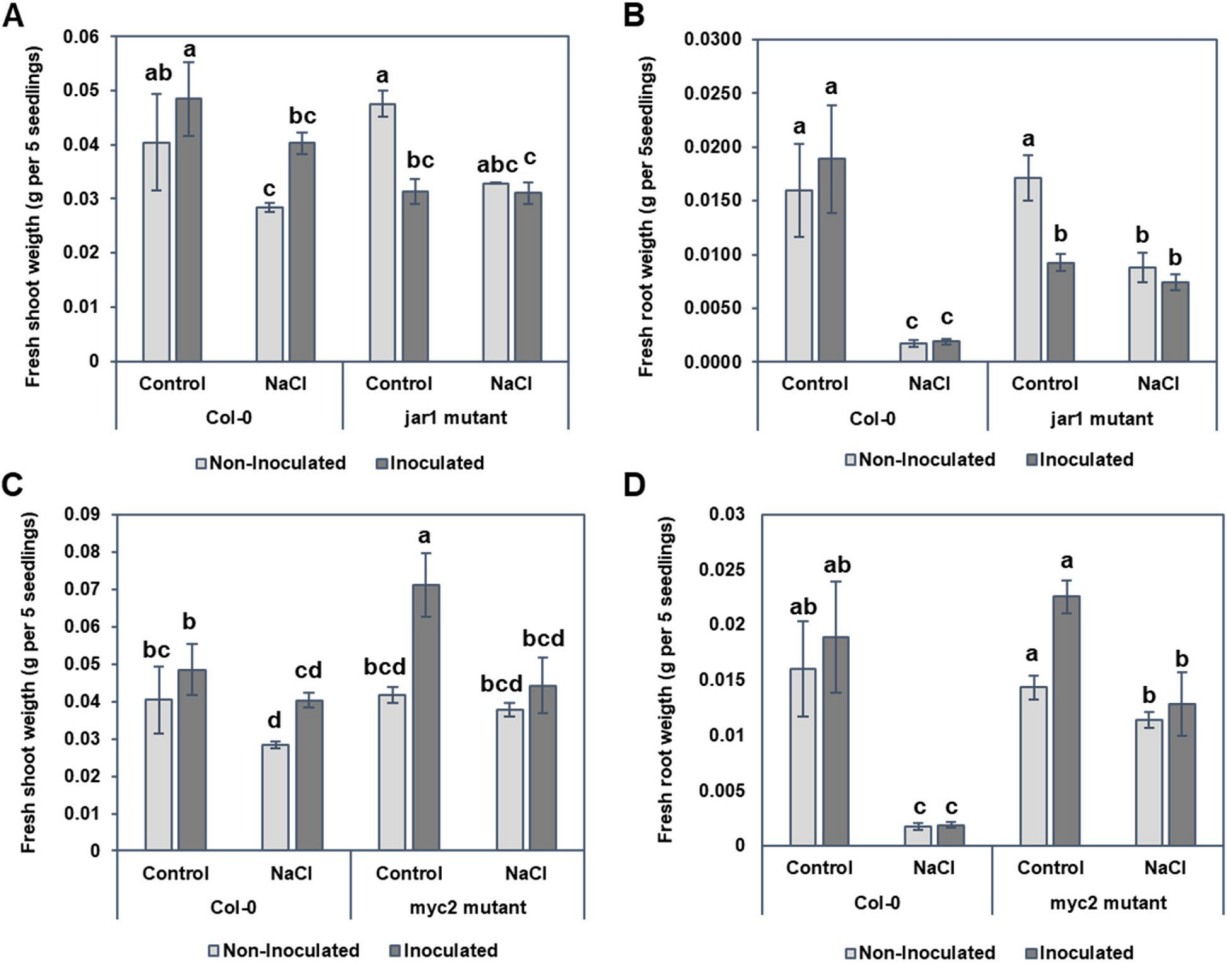
Shoot and root weight of *jar1* and *myc2* mutants compared with the Col-0 with and without *B. japonicum* under salt stress. **A** Shoot biomass of Col-0 and *jar1*, **B** root biomass of Col-0 and *jar1*, **C** shoot biomass of Col-0 and *myc2*, and **D** root biomass of Col-0 and *myc2.* Data are the mean ± standard error with different letters indicating significant differences. ANOVA-Tukey, *p* < 0.05

Although inoculation of Col-0 with B. japonicum increased shoot weight under salt and non-salt conditions, inoculation of *jar1* mutants with *B. japonicum* did not lead to significantly increased root or shoot growth under non-stressed conditions or any amelioration under salt stress (Fig. 6A and B). However, salt stress significantly reduced the fresh weight of Col-0 roots compared to those of *jar1*. This data indicates that *JAR1* plays a role in *B. japonicum*-induced growth under abiotic stress. In the shoots of myc2 mutants, inoculation with B. japonicum resulted in a trend similar to the control in non-stressed conditions. However, under salt stress, there was no significant difference between the inoculated and non-inoculated myc2 mutant plants (Fig. 6C). These data investigated that the PGPR-induced increase in biomass was absent in both *jar1* and myc2 plants, indicating that functional MYC2 and JAR1 are needed for *B. japonicum* to trigger the shoot biomass increase under salt stress.

### B. japonicum modulates JA-regulated gene expression during salt stress

**Fig. 7:**
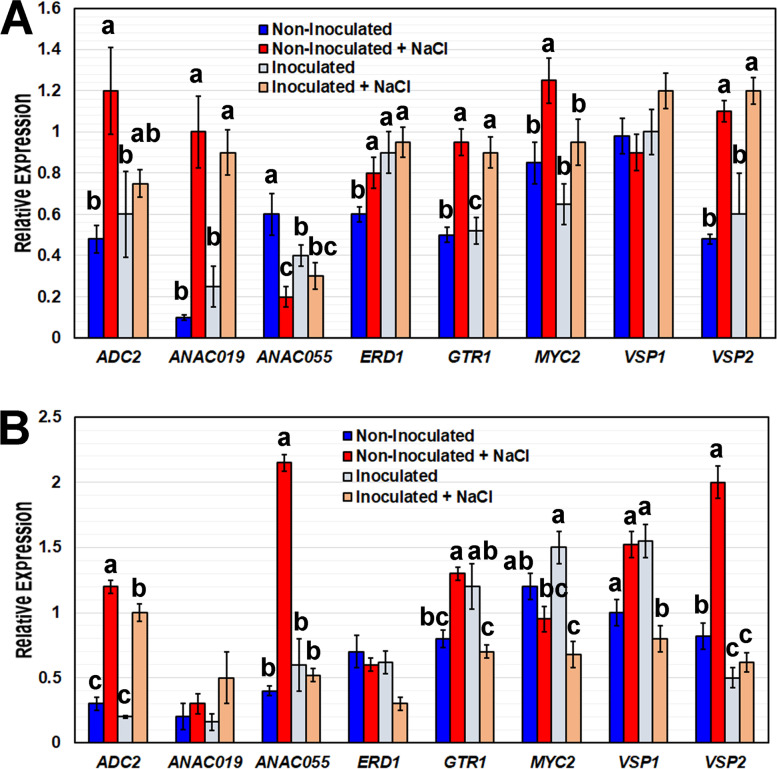
*B. japonicum* alters JA-regulated gene expression during salt stress. QRT-PCR analysis of JA-associated genes from non-inoculated and *B. japonicum*-inoculated tissue, with and without 100 mM NaCl treatment. Treatments were one week ± *B. japonicum* and 6 h ± 100 mM NaCl in (**A**) shoot and (**B**) root tissues. Mean expression relative to the *IPP2* reference gene ± standard error, with different letters representing significant differences separately for each gene, GLIM, Sequential Bonferroni, *p* < 0.05

Following the early activation of JA production in plants co-cultivated with *B. japonicum* and the likely consequent *B. japonicum*-activated priming, we measured expression of JA responsive genes under salt stress 6 h after starting the treatment. Plants were inoculated with the bacterium or control solution for one week prior to salt stress. Multiple JA-regulated genes were down-regulated by the bacterium in response to 100 mM NaCl. *Arginine Decarboxylase 2* (*ADC2*, At4g34710), *Dehydroascorbate Reductase* (*DHAR*, At1g19550), *Glutathione S-Transferase 1* (*GST1*, At1g02930), *Vegetative Storage Protein 1* (*VSP1*, At5g24780), *Vegetative Storage Protein 2* (*VSP2*, At5g24770) and an ATAF-like NAC-domain transcription factor (*ANAC055*, At3g15500) were reduced in root tissue and *DHAR* was reduced in shoots by *B. japonicum* relative to non-inoculated plants under salt stress (Fig. 7). Under salt stress, *MYC2* expression was reduced in shoot tissue but was not significantly altered in roots by *B. japonicum* compared to non-inoculated plants (Fig. 7). *ADC2*, known to be involved in salt tolerance [36], increased in shoots and roots in response to high salinity in non-inoculated plants, but was reduced as part of *B. japonicum*-influenced salt tolerance. *Early Response to Dehydration* (*ERD1*, At5g51070) and *Nac Domain Containing Protein 19* (*ANAC019*, At1g52890) expression levels were not altered by *B. japonicum* under salt stress (Fig. 7).

### B. japonicum differentially influences Response to Desiccation genes under salt stress

**Fig. 8:**
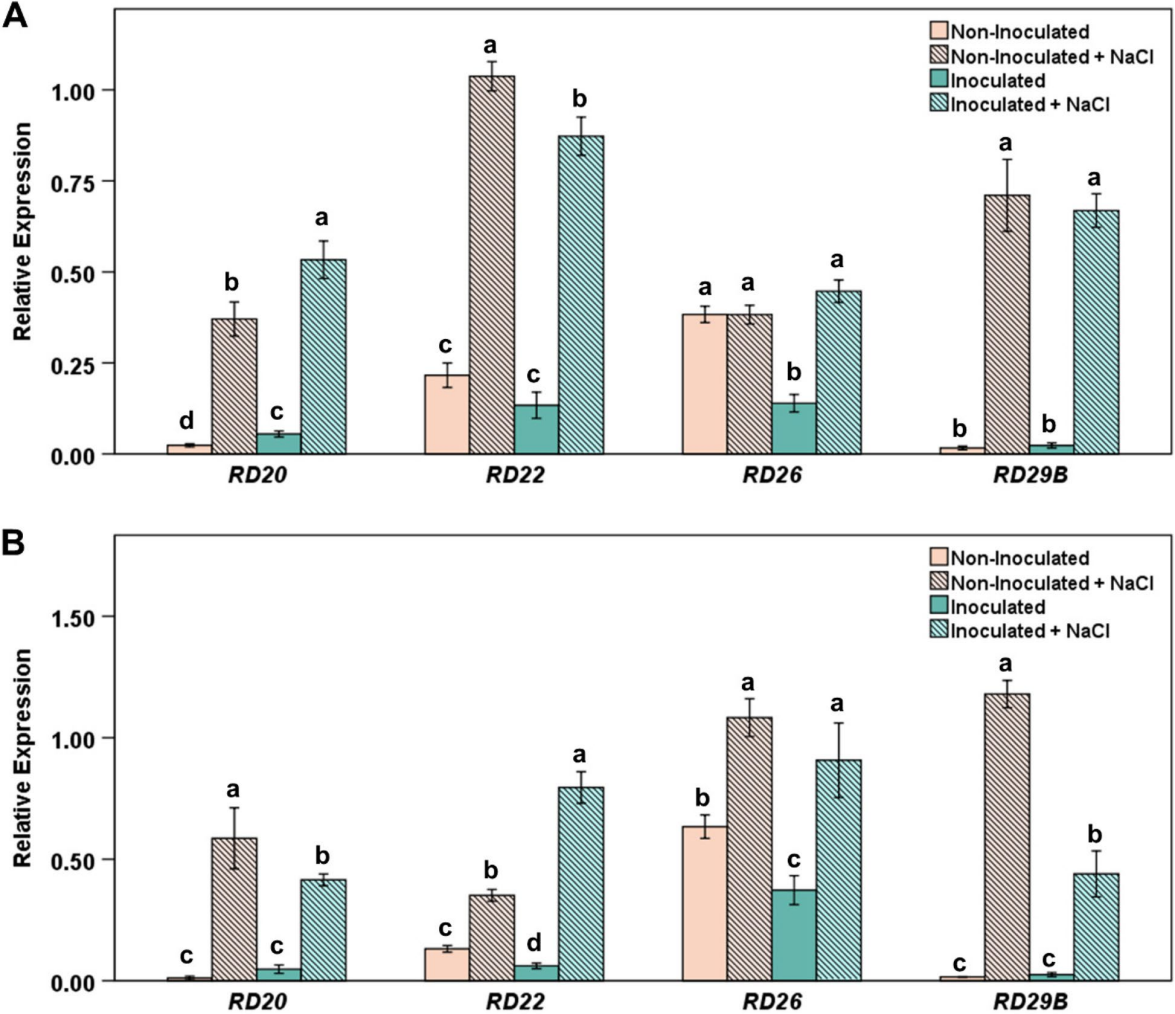
*B. japonicum* differentially stimulates *Response to Desiccation* genes during salt stress. Arabidopsis seedlings were treated with *B. japonicum* (inoculated) or 10 mM MgSO_4_ (non-inoculated) for seven days before being transferred to agar plates containing either no additional salt or 100 mM NaCl for six hours. (**A**, **B**) Graphs display qRT-PCR expression data relative to the *IPP2* reference gene from (**A**) shoot and (**B**) root tissues. Mean relative expression ± standard error with different letters representing significant differences, separately for each gene, ANOVA, *p* < 0.05

*Response to Desiccation* (*RD*) genes, *RD20* (At2g33380), *RD22* (At5g25610), *RD26* (At4g27410) and *RD29B* (At5g52300) have demonstrated responses to drought, salinity, ABA, and jasmonic acid [37–39]. In our study, salinity increased all four tested *RD* genes in roots and all but *RD26* in shoots without bacteria present (Fig. 8). *B. japonicum* differentially modulated salinity-induced *RD* gene expression, relative to non-inoculated salt-stressed plants. In inoculated salt-stressed plants, expression levels of *RD29B* and *RD20* were significantly reduced while expression of *RD22* was increased in the roots (Fig. 8B), and in shoots, *B. japonicum* enhanced *RD20* and decreased *RD22* expression (Fig. 8A and B). *RD22* expression is associated with drought and salt tolerance [40] and has been shown to be activated through *MYC2* [41]. A schematic diagram representing Arabidopsis JA-associated genes analyzed in this work for their potential roles in *B. japonicum*-stimulated salt tolerance is shown in Supplementary Figure S4.

## Discussion

### *B. japonicum* inhibits sodium accumulation and enhances shoot growth under salt stress in Arabidopsis

In this work, we used the interaction between *B. japonicum* and Arabidopsis to investigate the beneficial effects of PGPR to plants under salt stress. Previous studies have shown that salt stress reduces shoot and root development. In our study, *B. japonicum* significantly alleviated salt stress, returning shoot weight and MDA levels to those of the unstressed, uninoculated controls and limiting salt accumulation in the plant. Primary root length was shorter with *B. japonicum* co-cultivation [23] but no change was evident in the primary root length of *B. japonicum* inoculated plants with and without salt stress. Although we did not investigate the reason behind shortened primary root in *B. japonicum* inoculated plants under non-stress conditions in this study, we presume it to be due production of auxin by *B. japonicum* as discussed in our earlier investigation [23]. *B. japonicum* produces auxin and alters Arabidopsis root architecture through regulation of auxin efflux transporters PIN2, PIN3, PIN7 and ABCB19 [23]. However, under salt stress production of JA after *B. japonicum* inoculation may have played a role in the reduction of root weight, because exogenous application of methyl jasmonate (MeJA) under salt stress reduces root length and *jar1* mutants have longer roots and higher root fresh weight than mock plants [42]. Our results concur with the data obtained by Song et al. (2021) [42] showing the salt treatment significantly reduced the fresh weight of Col-0 compared to *jar1* (Fig. 6). Thus, indicating that JA is a negative regulator of root growth under salt stress. Since phytohormones, including JA and auxin, have been linked to PR length inhibition [23, 42, 43], more investigations are needed to decipher the crosstalk between the *B. japonicum* induced-auxin and JA under salt stress.

In order to maintain a balanced level of sodium ions, which is necessary for normal cellular functions, plants moderate the uptake and release of these ions in various ways [2]. Excess cytosolic sodium can have toxic effects on plant growth and reproduction [44]. In our data, measurements of sodium in the plants using Inductively Coupled Plasma-Optical Emission Spectrometry (ICP-OES) revealed that *B. japonicum* dramatically limited sodium accumulation in Arabidopsis. We hypothesized that the mechanisms used included sodium exclusion, priming or eventual ROS reduction/signaling.

Although JA is increased and is a suspected agent in *B. japonicum*-activated priming of Arabidopsis, multiple/various pathways were activated in the primed plants in response to salt stress. Using forward genetics, we focused on elucidating the mechanism through which *B. japonicum* confers salt tolerance to plants. Despite the overwhelming evidence that PGPR improve plant tolerance to abiotic stress, including salinity, the specific genetic mechanisms involved have remained elusive [45]. JA has been associated with salt tolerance in plants [46]. Exogenous jasmonate applications in the forms of seed priming, root drench and foliar spray have also been shown to improve multiple stress tolerance characteristics [47]. However, other studies have contrary results: exogenous application of methyl jasmonate decreased sodium uptake in salt-sensitive rice seedlings compared to salt-tolerant plants [48], and promoted the translocation of sodium ions from root to shoot in maize while concurrently reducing the amount of sodium ions in both roots and shoots [49]. According to Song et al. (2015), exogenous application of JA decreased salt stress tolerance, by enhancing H2O2 accumulation and reducing CAT2 expression, and jar1 mutant had increase salt tolerance [42] Song et al. (2015) showed that JA acts through a MYC-mediated repression of CAT2 expression. In rice, a JA mutant with lower JA accumulation than the wildtype, had higher salt tolerance (53). These contrasting conclusions about the role of JA show that researchers still have a long way to go in order to decipher complex interconnections involved in plant response to abiotic stress. Using PGPR adds another layer of complexity because they produce a myriad of metabolites and interact with multiple plant pathways. Although our study is not an exhaustive investigation of the mechanisms involved in the *B. japonicum*-*Arabidopsis* interaction under salt stress, it contributes to a better understanding of the role of PGPR in plant response to salinity.

### B. japonicum modulates jasmonate and hydrogen peroxide content toward priming in plants

*B. japonicum* activated jasmonate production in Arabidopsis as early as 0.5 hpi. The jasmonate group is composed of jasmonic acid (JA), its precursors, and its derivatives. JA-Ile is the biologically active form of JA that interacts with JAZ and COI1 proteins to result in activation of MYC transcription factors, including MYC2 [34, 50, 51]. JA 151, represents both endogenous and synthesized JA and JA 133 is specific to endogenous plant JA [52]. OPDA is necessary for the biosynthesis of biologically active JA-Ile, and it has also been shown to be critical to plant defense functions [53, 54]. In spite of the enhanced production of jasmonates in our study, no changes were observed in *JAR1* transcript levels and *MYC2* expression was not significantly up-regulated at 0.5 hpi. This is not surprising because in several PGPR-induced biological processes (e.g. ISR) that are regulated through JA, their inductions are not accompanied by increased JA production in the plants [21].

Studies have reported that co-cultivation with PGPR increases H_2_O_2_ content in the plants, which is a sign of the oxidative burst response and an aspect of PGPR priming [6, 10]. *B. japonicum* inoculation induced a transient increase in H_2_O_2_ within the first 0.5 h of co-cultivation and RBOH genes (responsible for ROS production) were upregulated at all measured time points (3—9 hpi). This early and temporal induction of ROS in biotic interactions, called oxidative burst, has been associated with biotic and abiotic stress tolerance in plants [51]. Both the increase in jasmonate production and the oxidative burst followed by a lowered basal H_2_O_2_ content are evidence of plant priming by *B. japonicum* [21, 55]. Primed plants demonstrate enhanced resistance to abiotic stress [16].

### B. japonicum differentially enhances oxidative stress responses in plants under high salinity

*B. japonicum* modulation of H_2_O_2_ upon inoculation led us to investigate *B. japonicum*-induced ROS production mediation of the plant response to salt stress. ROS production has been implicated in plant responses to pathogens, wounding, ABA, ozone, and heavy metals [56, 57]. Plant exposure to high salinity increases ROS levels [6, 9, 58]. Excessive cellular ROS causes oxidative stress, which, in turn, leads to lipid peroxidation, the oxidation of fatty acids by ROS. Malondialdehyde (MDA) is a byproduct of lipid peroxidation that serves as an indicator of membrane lipid damage caused by oxidative stress [59]. High concentrations of NaCl disrupt cellular functions and structures [45] through lipid peroxidation. In our study there was an increase in lipid peroxidation in plants subjected to salt stress, but pretreating the plants with *B. japonicum* lowered MDA levels under both the control and high salinity conditions.

For ROS to serve as a signaling molecule, its levels have to be tightly regulated, and ROS neutralization is a component of stress tolerance [4, 60]. Plants use a wide range of scavenging systems to maintain ROS homeostasis, including scavenging enzymes APX, SOD and CAT [6]. Among the early steps to regulate elevated ROS levels is the conjugation of superoxide (O_2_^−^) to H_2_O_2_ by superoxide dismutase (SOD) activity [6]. In wheat, jasmonates have been shown to increase SOD, CAT and APX in response to salt-induced oxidative stress, whereas salt treatment alone decreased them [61]. Endogenous JA in tomato activated ROS antioxidants in response to high salt [62]. A possible mechanism by which *B. japonicum* moderates the effects of stress, such as increased lipid peroxidation, may be by enhancing the antioxidant defense system. The *B. japonicum*-mediated basal H_2_O_2_ reduction at 3 dpi in Arabidopsis may involve ROS scavenging pathways. Few significant changes were present under non-salinity conditions between the inoculated and non-inoculated treatment. This may be because the gene expression analysis for genes encoding ROS scavenging enzymes was done six days after *B. japonicum* inoculation. However, our data clearly show a *B. japonicum-*induced modulation of H_2_O_2_ levels 0.5 hpi to 3 dpi.

### Plant stress-responsive genes, downstream of MYC2, are modulated by *B. japonicum* under salinity stress

JA has been shown to positively regulate salinity tolerance in plants [32, 43, 63]. Transgenic wheat and Arabidopsis plants constitutively expressing a JA synthesis gene displayed increased salt tolerance requiring functional *MYC2*, but not ABA synthesis [43]. *Pseudomonas putida* MTCC5279 ameliorated drought stress in chickpea plants by differentially modulating stress-responsive genes at different stages of drought stress, including slight down-regulation of *MYC2* [64]. *MYC2* regulates crosstalk between JA signaling and several phytohormones (ABA, IAA, SA and GA); participates in JA-regulated plant development; and is required for PGPR activated ISR [33]. *Pseudomonas* PS01 increased salt tolerance while up-regulating *LOX2* (a JA biosynthesis gene) and down-regulating *APX2* and *GLY17* in Arabidopsis under high salt conditions [32]. In our data, *MYC2* expression was elevated in *B. japonicum* inoculated plants at 0.5 hpi, although this was not statistically significant. However, in loss-of-function mutants *jar1* and *myc2*, inoculation with *B. japonicum* did not lead to the typical PGPR-induced increase in biomass both under salt stress and non-stress conditions indicating that *MYC2* and *JAR1* are essential for *B. japonicum* to promote growth and confer salt tolerance.

Treatment with *B. japonicum* did not increase ABA levels in the plants, yet it altered expression of several ABA-responsive genes such as *RD22, RD20, RD26, RD29B*, and *EDR1*. The effects on the ABA-responsive genes were more apparent after salt stress and we suspect that JA influenced the *B. japonicum*-induced regulation of these genes. Although ABA regulates the drought-induced expression of *MYC2* and *RD22*, ABA does not mediate *RD22* seed-specific activity [65], suggesting that other factors can also stimulate *RD22* expression [66]. RD20, a calcium binding protein, is induced by ABA and has roles in oxylipin metabolism during abiotic stress [67]. *RD26* overexpression plants highly express *RD20* [39] and *RD26* targets *RD20* [68]. Although *RD26* has been primarily linked to an ABA dependent pathway, in ABA-deficient mutants (*aba2*) under salt stress, *RD26* was highly induced, suggesting its involvement in a separate pathway during salt stress [39]. *RD26* has also been shown to bind and activate the *ERD1* promoter, a jasmonic acid induced stress gene [38, 69]. Jasmonic acid has been shown to influence *RD29B* through priming [70] and under abiotic stress through the *CBF/DREB1* transcription factor [71]. In abiotic stress conditions, *MYC2* contributes to the activation of *RD29B* [70], although whether this *MYC2*-induced transcription is due to an ABA-dependent pathway or stress-related JA pathways is still unclear. The association of *RD20*, *RD22*, *RD26*, and *RD29B* with the JA pathway under abiotic stress [37, 38, 70] suggests that *B. japonicum* most likely induces plant salt tolerance through the JA pathway.

Soil microbes are known to trigger and manipulate plant mechanisms, including hormone signaling, to enhance salt stress tolerance in plants [72]. Although increases in ABA are often seen in plants under salt stress, not all PGPR promote plant ABA production. Under high salinity, *Bacillus amyloliquefaciens* SQR9 was found to reduce maize ABA content to the no-stress level [73]. *B. amyloliquefaciens* FZB42 induced salt tolerance in Arabidopsis likely by activating plant ET/JA signaling but not ABA-dependent pathways [63]. MYC2 acts as an activator or as a repressor to regulate JA responses [74].

Different PGPR use unique stress resistance strategies. Pinedo and coworkers found that *Burkholderia phytofirmans* PsJN differentially induced transcriptomic changes in Arabidopsis *RD29A, RD29B, APX2, GLY17, PDF1.2* and *LOX2* over time, especially in roots, under salt stress [10]. Hormonal crosstalk between multiple phytohormone signaling pathways likely produced the complex response levels and timing required to enhance salt tolerance [10]. Abiotic stress such as salinity and drought increase ethylene levels in plants through induction of a key enzyme, 1-aminocyclopropane-1-carboxylate synthase (ACC synthase), in ethylene biosynthesis. High levels of ethylene result in plant damage, limit nutrient uptake and inhibit root growth [75]. PGPR produced ACC-deaminase can hydrolyze ACC to ammonia and a-ketobutyrate, thereby reducing the concentration of ethylene in the plants and alleviating ethylene-induced stress [75] For example, the PGPR *Enterobacter* sp. UPMR18 was found to improve salinity tolerance in okra, with the help of the bacterium’s ACC deaminase capability as well as by influencing increased ROS scavenging activity in plants [76].

PGPR have been shown to increase [77] and decrease [32] ROS scavenging activity in plants toward enhanced salt tolerance [9]. *Bacillus amyloliquefaciens* FZB42 volatile compounds promoted salt tolerance through up-regulation of *POD, CAT*, *SOD* and JA synthesis in Arabidopsis [78].

## Conclusion

Our results suggest that *B. japonicum* confers salt tolerance through a JA-regulated pathway under abiotic stress. Our results contribute to understanding how soil microbes improve plant tolerance to salt stress and open doors to the possibilities of managing salinity stress using soil microbiota.

## Methods

### Plant material and growth conditions

All plants used in this study were in the *Arabidopsis thaliana*, Columbia (Col-0) background. Col-0 seeds were obtained from the Arabidopsis Biological Resource Center (ABRC), while m*yc2* (aka *jin1*) (ABRC stock number CS13115) and *jar1*(ABRC stock number CS8072) seeds were kindly provided by Thomas Eulgem [79]. Plants were grown vertically, with a twelve/twelve hour (h) day/night cycle, under 120–150 μmol m^−2^ s^−1^ light, at 22 °C. Seeds were surface sterilized (three minutes [min] in 10% [v/v] NaClO and 0.05% Tween 80 solution, one min in 70% EtOH, four sterile water rinses) and cold treated at 4 °C for two days before being sown onto a germination medium (1% [w/v] agar [Fisher Scientific], half-strength Murashige and Skoog [½ MS] supplemented with 0.5% sucrose, 0.01% myo-inositol and 0.05% MES, pH 5.7). After four days, uniform seedlings were transferred to a treatment medium (1% agar, ½ MS, pH 5.7), eight to ten seedlings per square Petri dish, for each treatment period. Shoots and roots were weighed after seven days of salt stress treatment.

### Bradyrhizobium japonicum IRAT FA3 cultivation and plant inoculation

*B. japonicum* IRAT FA3 was cultured in half strength Luria–Bertani (LB) liquid medium at 28 °C on a shaker at 100 rpm. Bacterial inoculum was prepared after 24 h of growth by centrifugation of bacterial cell culture for ten min at 1,500 rpm followed by resuspension of bacterial cells in sterile 10 mM MgSO_4_. The bacterial titer was adjusted to 1.0 optical density at 600 nm (1.8 × 10^9^ colony-forming units mL^−1^). To treat plants with the bacteria, ten microliters of inoculum were applied onto the roots of vertically grown four-day-old seedlings on agar.

### Primary root growth assay

Seedlings, at four days old, were treated with or without PGPR and placed on half-strength Murashige and Skoog media with or without 100 mM NaCl. Initial primary root measurements were taken, the total primary root lengths of plants were measured ten days after inoculation, and new growth after inoculation and salt stress was calculated. Plants were photographed ten days after inoculation. All experiments were repeated at least three times. Each treatment contained six replicates with eight-ten plants per replicate.

### Stress treatments

To determine an appropriate salt concentration, four-day-old seedlings were placed on half strength MS plates amended with a concentration of 0, 50, 100, or 200 mM NaCl. Shoot and root weights were measured fourteen days after stress application. One of the salt concentrations (100 mM) was chosen for stress experiments based on shoot weight results. Each treatment contained six replicates with eight-ten plants per replicate. Salt stress was applied seven days after inoculation with *B. japonicum*, except in the case of primary root growth experiments.

### Sodium, carbon, and nitrogen content determination

Plants were collected after seven days of salt stress application and fourteen total days of *B. japonicum* treatment, briefly rinsed, blotted dry, placed in 50 mL Falcon tubes, and placed in a drying oven at 104°F for four days. Samples were transferred to two milliliter round bottom tubes, ground with beads, and analyzed by the Environmental Sciences Research Laboratory (ESRL) at the University of California, Riverside. For sodium content, samples were digested using an Anton-Paar microwave assisted digestion process. Digestion was done using a one:three ratio of nitric and hydrochloric acid. Measurements were done using an Inductively Coupled Plasma-Optical Emission Spectrometry (ICP-OES) system.

### Root colonization assay under stress

Arabidopsis plants were grown on MS-agar plates and subjected to stress as described above. After 10 days of co-cultivation, one cm root pieces were cut from the root using a sterile razor blade. Roots from ten plants were combined into one replicate. Roots were washed in sterile dH_2_O twice and then placed in 100 µl of phosphate buffered saline (137 mM NaCl, 10 mM Na_2_HPO_4_, 2.7 mM KCl, 2 mM KH_2_PO_4_) as performed by Allard-Massicote et al., (2016) [80]. The roots in PBS were sonicated for a total of ten—thirty second pulses on a low setting in an ultrasonic bath (CPX3800, Fisherbrand). Ten microliters of a 10^–3^ dilution of each sonicated solution were plated on half strength LB agar plates. Colonies were counted after two to three days of incubation at 28℃. This was replicated six times with three technical replicates per biological replicate. The averages of the technical replicates were used for statistical analysis.

### B. japonicum growth under stress

*B. japonicum* was grown in liquid culture composed of tryptone 10 g/L, yeast extract 5 g/L, and a final sodium chloride concentration of 0 mM, 50 mM 100 mM, 200 mM or 300 mM and incubated at 28 °C at 100 rpm. The optical density at 600 nm was measured after 24 h.

### RNA isolation and reverse transcription–qRT-PCR

Arabidopsis shoot and root tissues (30–50 mg) were separated and collected from plants for RNA extraction six hours after salt stress application. Total RNA extraction was performed using GeneJet plant RNA purification kit (ThermoScientific, K082) according to the manufacturer's instructions. First-strand cDNA was synthesized from two μg of total RNA by using the Revert Aid™ H Minus First Strand cDNA synthesis kit (ThermoScientific, K1622) according to the instructions of the manufacturer and treated with DNase I (ThermoScientific, EN0521). Gene-specific primers used in the qRT-PCR reaction to determine gene expression are listed in Table 1. Quantitative real-time PCR analysis was performed using Maxima SYBR Green qPCR MasterMix (2x) (ThermoScientific, K0252) on a Biorad Connect CFX system. Expression of all genes was normalized relative to the *IPP2* reference gene. All experiments were done in triplicate and repeated three times.

**Table 1:** q(RT)PCR primers

Gene	Forward	Reverse	Reference
*CAT3*	TCACAGCCACGCCACTAA	AGAACCAAGCGACCAACC	[81]
*MSD1*	GAAGAAGCTAGTTGTTGACAC	CCTCGCTTGCATATTTCCAG	[81]
*APX1*	TTTCCACCCTGGAAGAGAGGAC	TGGTCACAACCCTTGGTAGCATC	[5]
*MDAR2*	AACCAAGGTGTCAAGCCTGGTG	AGGACGCTCATATGGAGGAACTTG	[5]
*RBOHD*	ACTCTCCGCTGATTCCAACG	ATCGCCGGAGACGTTATTCC	[82]
*RBOHE*	AAGACCTCGTCATGTGGTTCA	AGAACCCAGCTTCTTTGCCA	[82]
*RBOHF*	CTTGGCATTGGTGCAACTCC	TCTTTCGTCTTGGCGTGTCA	[82]
*JAR1*	TCGGTCCGTACTGCAATGTC	GCGGGAATCAATCCGTACCA	[83]
*MYC2*	GTCACCGGTTTATGGAATGA	CGGAAGAGCTCATGAAAGC	[34]
*ADC2*	GGACTAAGCATTCGGGTCAT	TTCCTCACCACACGAACAAT	[34]
*ANAC019*	TTCTCGTAGAAACGGAAGCA	ACGAAGTACCGTTGTTGCTG	[83]
*ANAC055*	AACCGGGTTTCAGGGTTTAG	CACCGTCACCGTAACGAATA	[83]
*DHAR*	CGGCGACTGTCCGTTCAG	TCAGATGGATTTTGTAGGTAAGACTCTT	[84]
*ERD1*	TTTCACCATTGGAGGATTG	TACCTCACGGACTAATCAC	[85]
*GTR1-1*	GTCCATTGGCTGGTATTGCT	ACTTGCTGCAACGTGCATAG	[86]
*VSP1*	GGACTTGCCCTAAAGAACGA	GTGTTCTCGGTCCCATATCC	[34]
*VSP2*	CCTAAAGAACGACACCGTCA	TCGGTCTTCTCTGTTCCGTA	[34]
*RD20*	TTTGGACCTTACTCATAAACTTAGC	TTAGTCTTGTTTGCGAGAATTGGC	[87]
*RD22*	ACCATTGAGGAGTGTGAAGCCAG	CTAGTAGCTGAACCACACAACATGAG	[87]
*RD26*	AGGTCTTAATCCAATTCCAGAGCTA	ACCCATCAGTAACTTCACATCTCTC	[88]
*RD29B*	AAGAACGTCGTTGCCTCA	GCCCGTAAGCAGTAACAG	[87]
*IPP2*	CTCCCTTGGGACGTATGCTG	TTGAACCTTCACGTCTCGCA	[89]

### Lipid peroxidation assay

Approximately thirty mg of shoot tissue was collected after seven days of salt stress, ground in liquid nitrogen, and used for quantification. Lipid peroxidation was determined using the thiobarbituric acid (TBA) test as described by Hodges et al. (1999) and Zhao et al. (2017) [25, 90]. Absorbances were read with a spectrophotometer at 440, 532, and 600 nm. The MDA contents were calculated using the formula below:

MDA equivalents (nmol mL^−1^) = 1 [(A-B))/157 000] × 10 ^6^ where *A* = [(Abs 532_RSII_–Abs 600_RSII_)] and *B* = [(Abs 440_RSII_–Abs600_RSII_) × 0.0571], with Abs as absorbance using reagent solution II (RSII). MDA equivalents (nmol g^−1^ FW) = MDA equivalents (nmol mL^−1^) x total volume of the extracts (mL) / g FW or number of seedlings. Reagent solution I (RSI) is 20% (w/v) TCA and 0.01% butylated hydroxytoluene (BHT), and reagent solution II (RSII) is RSI and 0.65% (w/v) 2-thiobarbituric acid (TBA). TCA was used as the reference solution.

### Determination of chlorophyll content

Chlorophyll content was determined according to Zhao et al. (2017) [80]. Approximately 30 mg of shoot tissue were collected after seven days of salt stress, ground in liquid nitrogen, and used for quantification. The tissue was homogenized in two mL 80% (v/v) aqueous acetone and incubated under shaking conditions at room temperature for thirty min in the dark. Absorbance of the supernatant obtained after centrifugation at 12,000 g for five min at room temperature was determined at 663 and 645 nm. Chlorophyll content was determined using the formula below:

$$C (mg g-1 FW)\hspace{0.17em}=\hspace{0.17em}0.002 x (20.2\hspace{0.17em}\times \hspace{0.17em}OD645\hspace{0.17em}+\hspace{0.17em}8.02\hspace{0.17em}\times \hspace{0.17em}OD663)/g FW$$

where C represents the total chlorophyll (a + b) content.

### Hydrogen peroxide measurement

At fourteen days-old, plants were inoculated and harvested at 0.5 h, 3 h, 6 h, and 12 h and three days post inoculation. Shoot and root tissues were separated at collection. Samples were frozen, weighed, and kept on dry ice in the dark until the hydrogen peroxide assay was performed. Once all samples were collected, the tissue was ground and the appropriate amount of ice cold 20 mM K_2_HPO_4_ buffer was added to each sample according to Le et al. (2016) [91]. Hydrogen peroxide quantification was performed using the Amplex Red Hydrogen Peroxide/Peroxidase Assay Kit (Invitrogen A22188) according to the manufacturer’s instructions. The assay was performed using three biological replicates and three technical replicates. Fluorescence was measured at 525 nm using a Promega Glomax MultiDetection System with a Green Optical Kit.

### Quantification of jasmonic acid and abscisic acid

Arabidopsis plants were inoculated with *B. japonicum* and harvested at 0, 0.5, 1, 3, and 6 h after inoculation. Whole plants were frozen in liquid nitrogen, then freeze-dried, and fifty mg of tissue were homogenized; ten mg of which were used for each sample. Ten replicates, each consisting of approximately ten to twelve plates, were used in statistical analyses. Jasmonic acid and abscisic acid were extracted and quantified using ultra performance liquid chromatography-mass spectrometry by the Metabolomics Core Facility at the University of California, Riverside according to Sheflin et al. (2019) [92].

### Statistical analysis

All experiments consisted of at least three biological replicates with the mean and standard error shown in figures. SPSS Statistics v. 26 (IBM) was used to make all figures and conduct all statistical analyses. Data that fit ANOVA assumptions were analyzed using an ANOVA and Tukey’s Honest Significant Difference post-hoc test. Data that did not fit these assumptions were analyzed using the normal Generalized Linear Model with the identity link function and Sequential Bonferroni correction.  

## Supplementary Information

**Additional file 1:** **Supplementary Fig. S1. **Determination of salt stress experimentalparameters by measuring effects on Arabidopsis shoot and root weight. (**A**) Shoot and (**B**) root fresh weights were measured 14 days after the addition of 0mM (control), 50 mM, 100 mM or 200 mM NaCl stress treatment. Data are the means ± standard error with different lettersindicating significant differences. ANOVA; Tukey, *p* < 0.05.  Null seedlings did not survive treatment. **Supplemental Fig. S2.** Growth of *B. japonicum *under salt treatment. (**A**)Growth of *B. japonicum* in halfstrength Luria Broth without NaCl and supplementation with increasingNaCl concentrations compared to the  commercially manufactured rate (½ LB) was determined after 24 hours. (**B**) Quantificationof root colonization by *B. japonicum*under 100 mM salinity stress after 10 days of inoculation and stress treatment.(**A, B**) Data are mean colony forming units (CFU) ± standard error for 6experimental replicates. Letters indicate significant differences. (**A**) ANOVA; Tukey, *p* < 0.05. (**B**) Student’s *t*-test; *p* < 0.05 No significant differenceswere found. **Supplementary Fig. S3.** Abscisic acid (ABA) production in *A. thaliana *under *B.japonicum* inoculation.*A. thaliana* seedlingswere treated with *B. japonicum*(inoculated)or 10 mM MgSO_4_(non-inoculated) for up to 6 hours. ABA levels were measured by liquidchromatography mass-spectrometry (LCMS). Graphs show the means ± standarderror. No significant differences were seen between inoculated andnon-inoculated treatments at each timepoint. Student’s t-test, *p* < 0.05. **Supplemental Fig. S4. **Schematic diagramrepresenting *A. thaliana*JA-associated genes analyzed in *B.japonicum*inoculated salt treated plants.Lines indicate known signaling pathways under abiotic stress. Dashedlinesdenote putative induction by *B. japonicum.*

## Data Availability

All data generated and/or analyzed during this study are available from the corresponding author on reasonable request. No sequencing, genomic, or phylogenetic data were generated during this study.
