# Supplementary material for: Bradyrhizobium japonicum IRAT FA3 promotes salt tolerance through jasmonic acid priming in Arabidopsis thaliana
Source: BMC Plant Biol. 2023 Jan 30;23:60. doi: 10.1186/s12870-022-03977-z (PMC9885586; doi:10.1186/s12870-022-03977-z)
Supplement: Supplementary file 1 — Additional file 1: Supplementary Fig. S1. Determination of salt stress experimentalparameters by measuring effects on Arabidopsis shoot and root weight. (A) Shoot and (B) root fresh weights were measured 14 days after the addition of 0mM (control), 50 mM, 100 mM or 200 mM NaCl stress treatment. Data are the means ± standard error with different lettersindicating significant differences. ANOVA; Tukey, p < 0.05. Null seedlings did not survive treatment. Supplemental Fig. S2. Growth of B. japonicum under salt treatment. (A)Growth of B. japonicum in halfstrength Luria Broth without NaCl and supplementation with increasingNaCl concentrations compared to the commercially manufactured rate (½ LB) was determined after 24 hours. (B) Quantificationof root colonization by B. japonicumunder 100 mM salinity stress after 10 days of inoculation and stress treatment.(A, B) Data are mean colony forming units (CFU) ± standard error for 6experimental replicates. Letters indicate significant differences. (A) ANOVA; Tukey, p < 0.05. (B) Student’s t-test; p < 0.05 No significant differenceswere found. Supplementary Fig. S3. Abscisic acid (ABA) production in A. thaliana under B.japonicum inoculation.A. thaliana seedlingswere treated with B. japonicum(inoculated)or 10 mM MgSO4(non-inoculated) for up to 6 hours. ABA levels were measured by liquidchromatography mass-spectrometry (LCMS). Graphs show the means ± standarderror. No significant differences were seen between inoculated andnon-inoculated treatments at each timepoint. Student’s t-test, p < 0.05. Supplemental Fig. S4. Schematic diagramrepresenting A. thalianaJA-associated genes analyzed in B.japonicuminoculated salt treated plants.Lines indicate known signaling pathways under abiotic stress. Dashedlinesdenote putative induction by B. japonicum. [file 12870_2022_3977_MOESM1_ESM.docx]

**SUPPLEMENTARY MATERIAL**

***Bradyrhizobium japonicum* IRAT FA3 promotes salt tolerance through jasmonic acid priming in *Arabidopsis thaliana***

Melissa Y. Gomez, Mercedes M. Schroeder, Nathan K. McLain, Caovinh Le, Nguyen C. Tran, and Emma W. Gachomo


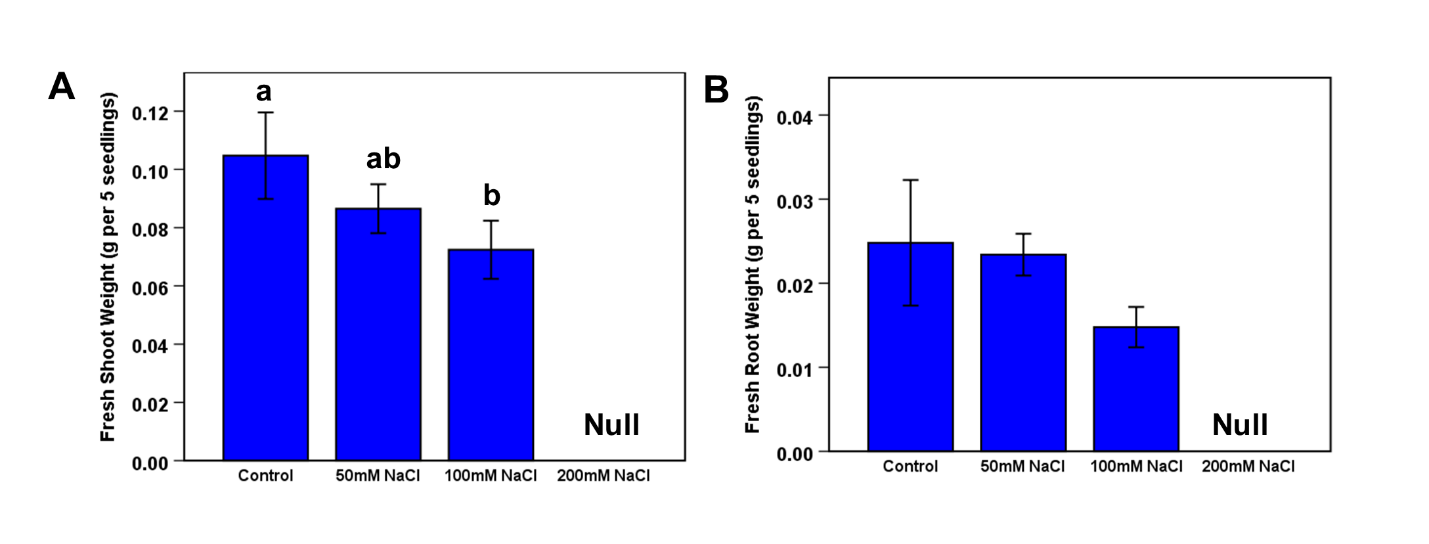


**Supplementary Fig. S1.** Determination of salt stress experimental parameters by measuring effects on Arabidopsis shoot and root weight. (**A**) Shoot and (**B**) root fresh weights were measured 14 days after the addition of 0 mM (control), 50 mM, 100 mM or 200 mM NaCl stress treatment. Data are the means ± standard error with different letters indicating significant differences. ANOVA; Tukey, *p* < 0.05.  Null seedlings did not survive treatment.


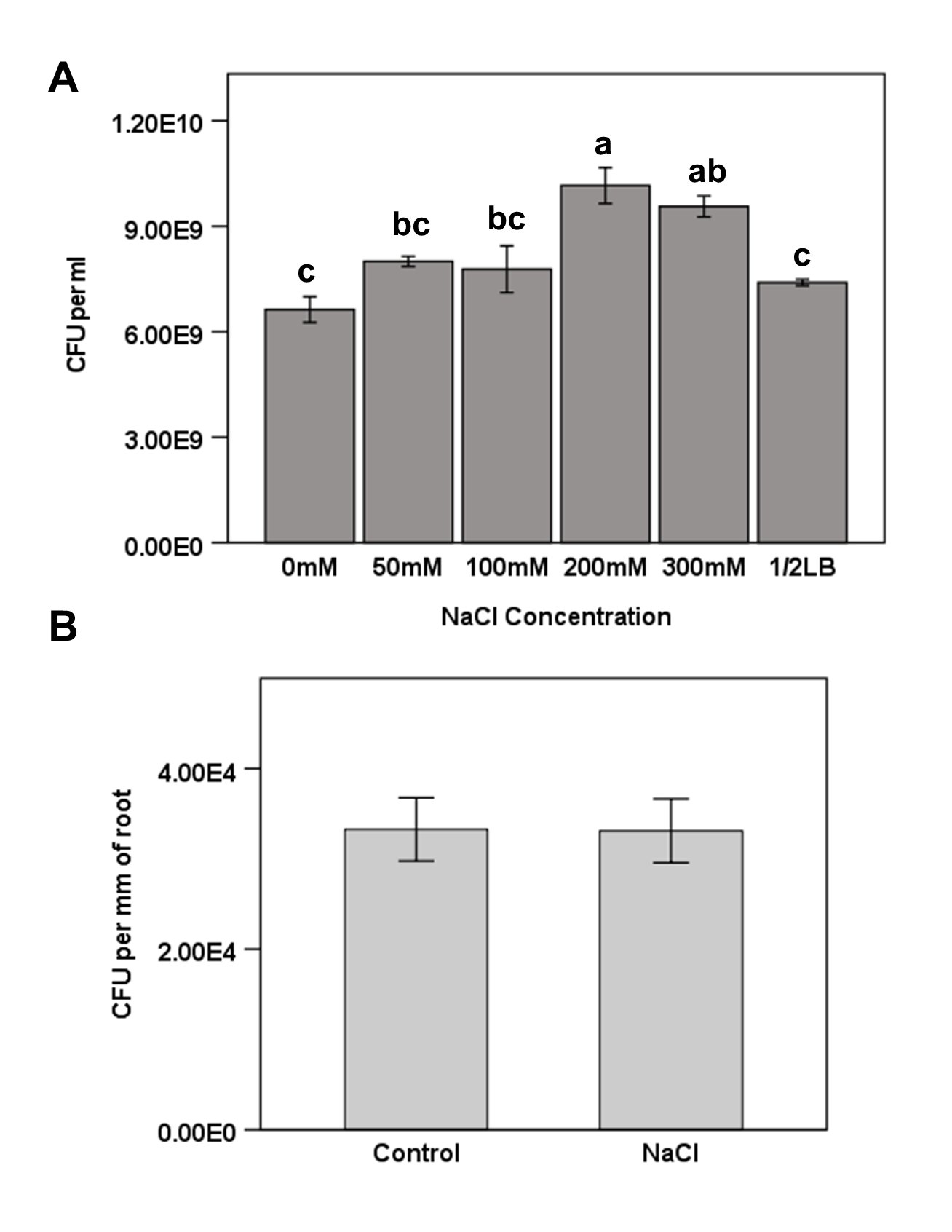


**Supplemental Fig. S2.** Growth of *B. japonicum* under salt treatment. (**A**) Growth of *B. japonicum* in half strength Luria Broth without NaCl and supplementation with increasing NaCl concentrations compared to the commercially manufactured rate (½ LB) was determined after 24 hours. (**B**) Quantification of root colonization by *B. japonicum* under 100 mM salinity stress after 10 days of inoculation and stress treatment. (**A, B**) Data are mean colony forming units (CFU) ± standard error for 6 experimental replicates. Letters indicate significant differences. (**A**) ANOVA; Tukey, *p* < 0.05. (**B**) Student’s *t*-test; *p* < 0.05 No significant differences were found.

**
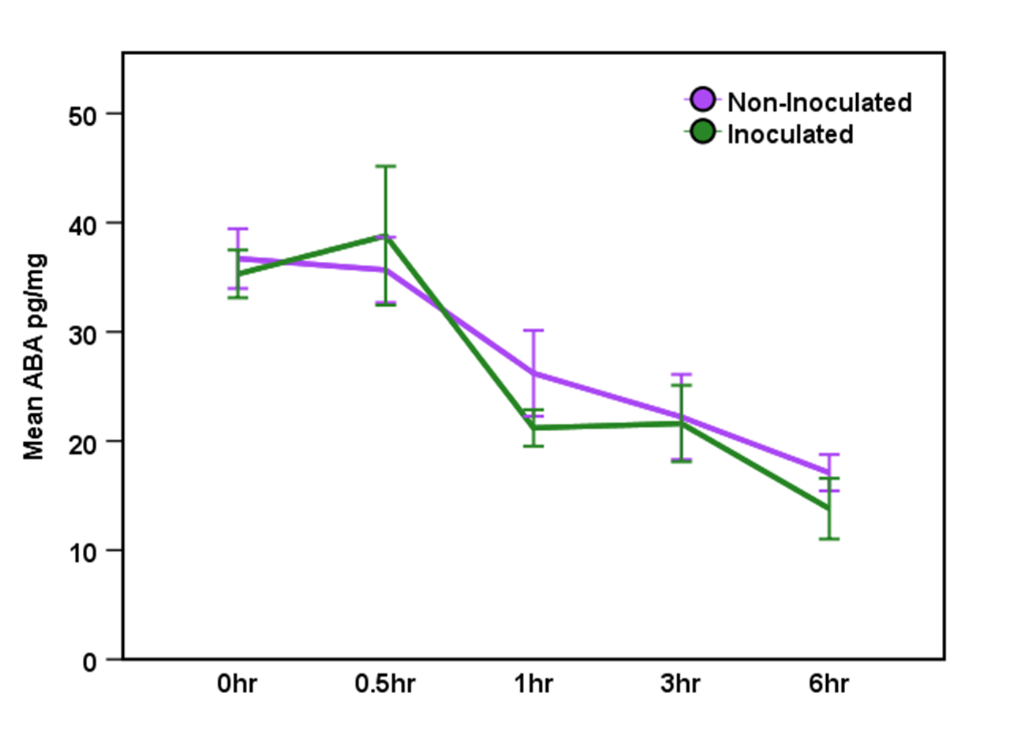
**

**Supplementary Fig. S3.** Abscisic acid (ABA) production in *A. thaliana* under *B. japonicum* inoculation. *A. thaliana* seedlings were treated with *B. japonicum* (inoculated) or 10 mM MgSO_4_ (non-inoculated) for up to 6 hours. ABA levels were measured by liquid chromatography mass-spectrometry (LCMS). Graphs show the means ± standard error. No significant differences were seen between inoculated and non-inoculated treatments at each timepoint. Student’s t-test, p < 0.05.


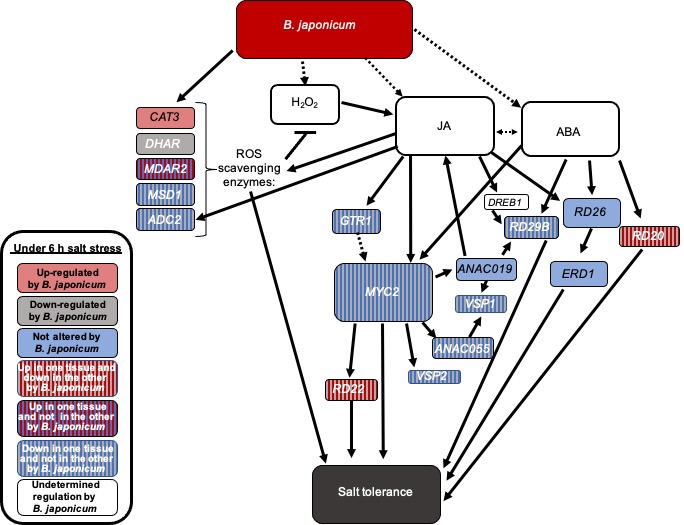


**Supplemental Fig. S4.** Schematic diagram representing *A. thaliana* JA-associated genes analyzed in *B. japonicum*inoculated salt treated plants. Lines indicate known signaling pathways under abiotic stress. Dashed linesdenote putative induction by *B. japonicum*.
